# Phenylpropanoid Biosynthesis Gene Expression Precedes Lignin Accumulation During Shoot Development in Lowland and Upland Switchgrass Genotypes

**DOI:** 10.3389/fpls.2021.640930

**Published:** 2021-08-09

**Authors:** Prasenjit Saha, Fan Lin, Sandra Thibivilliers, Yi Xiong, Chongle Pan, Laura E. Bartley

**Affiliations:** ^1^Department of Microbiology and Plant Biology, University of Oklahoma, Norman, OK, United States; ^2^School of Computer Science, University of Oklahoma, Norman, OK, United States; ^3^Research Institute for the Sustainable Humanosphere, Kyoto University, Kyoto, Japan; ^4^Institute of Biological Chemistry, Washington State University, Pullman, WA, United States

**Keywords:** biomass, cell wall, digestibility, lignin, ferulic acid, switchgrass, vegetative development

## Abstract

Efficient conversion of lignocellulosic biomass into biofuels is influenced by biomass composition and structure. Lignin and other cell wall phenylpropanoids, such as *para*-coumaric acid (*p*CA) and ferulic acid (FA), reduce cell wall sugar accessibility and hamper biochemical fuel production. Toward identifying the timing and key parameters of cell wall recalcitrance across different switchgrass genotypes, this study measured cell wall composition and lignin biosynthesis gene expression in three switchgrass genotypes, A4 and AP13, representing the lowland ecotype, and VS16, representing the upland ecotype, at three developmental stages [Vegetative 3 (V3), Elongation 4 (E4), and Reproductive 3 (R3)] and three segments (S1–S3) of the E4 stage under greenhouse conditions. A decrease in cell wall digestibility and an increase in phenylpropanoids occur across development. Compared with AP13 and A4, VS16 has significantly less lignin and greater cell wall digestibility at the V3 and E4 stages; however, differences among genotypes diminish by the R3 stage. Gini correlation analysis across all genotypes revealed that lignin and *p*CA, but also pectin monosaccharide components, show the greatest negative correlations with digestibility. Lignin and *p*CA accumulation is delayed compared with expression of phenylpropanoid biosynthesis genes, while FA accumulation coincides with expression of these genes. The different cell wall component accumulation profiles and gene expression correlations may have implications for system biology approaches to identify additional gene products with cell wall component synthesis and regulation functions.

## Introduction

Due to increased transportation energy usage and urgency to reduce fossil fuel use, demand for advanced fuels is predicted to increase to 79.5 billion liters by 2022 (US CRS Report, 2009), about 7% of annual petroleum utilization (EIA, 2019). Biofuels from lignocellulosic biomass, such as the leaves and stems of perennial grasses, hold promise to sustainably fulfill a significant fraction of the alternative fuel requirement with low greenhouse gas emissions (Schmer et al., 2008; Gelfand et al., 2013). Biochemical processes are now being deployed that convert polysaccharides, typically cellulose, from plant cell walls into alcohol fuels (Youngs and Somerville, 2012; Torres et al., 2016). However, lignin and hydroxycinnamic acids (HCAs) covalently crosslink cell walls and reduce saccharification efficiency during biomass enzymatic digestibility (ED) (Sattler and Funnell-Harris, 2013). Altering expression of single genes, especially those from the phenylpropanoid biosynthesis pathway, which synthesizes lignin and HCAs, improves biomass processing efficiency (Baxter et al., 2014; Li et al., 2018). Still, questions remain as to which manipulations are optimal and how genetic diversity can be harnessed to achieve simultaneous biomass composition and yield improvements. One approach to address this is to associate transcriptomes with biomass properties at harvest, but designing such studies requires an initial understanding of the relationships between gene expression (GE) and composition across development and genotypes. This study aims to reduce this knowledge gap for switchgrass.

Among potential dedicated bioenergy grasses, switchgrass (*Panicum virgatum* L.) is a front-runner species for lignocellulosic feedstock production in the United States (Bouton, 2007; Casler et al., 2011; Bartley et al., 2013b). Switchgrass is a C4, warm-season perennial that produces high annual biomass yield (typically ≥12 Mg/ha) and exhibits broad environmental adaptation (Lowry et al., 2019). This primarily outcrossing species consists of upland and lowland ecotypes and possesses high genetic diversity (Zalapa et al., 2011; Lu et al., 2013). Lowland genotypes are typically tetraploid, whereas uplands are octaploid or tetraploid (Lu et al., 2013). Most switchgrass cultivars have only undergone a few rounds of selection, and more genetic diversity exists within a cultivar than among cultivars (Cortese et al., 2010). That said, there are established characteristics that typify the ecotypes. Relative to upland cultivars under the same conditions, lowlands tend to cease growth later and have longer, thicker stems, contributing to lowlands typically accumulating greater biomass than uplands (Lowry et al., 2014). On the other hand, uplands exhibit greater drought and cold tolerance than lowlands (Stroup et al., 2003; Ayyappan et al., 2017).

The relationships between GE and cell wall properties across development and among ecotypes and genotypes remain under-explored. Generally, as plants mature, secondary cell wall formation and lignification occur; as a result, mature tissue contains a higher proportion of lignin and is less digestible (Boerjan et al., 2003). Previous cell wall and GE analyses revealed variations among developmental stages and internodes from lowland switchgrass of the Alamo cultivar (Mann et al., 2009; Shen et al., 2009; Escamilla-Treviño et al., 2010; Shen et al., 2013). Cell wall digestibility, a key output of biomass composition, varies across switchgrass development due to changes in cell wall components, with strong negative correlations between digestibility and total lignin, lignin monomers, and HCAs in Alamo switchgrass (Shen et al., 2009; Hu et al., 2010). However, whether developmentally associated cell wall changes are consistent among genotypes has not to our knowledge been examined. When genotypic variation effects on switchgrass cell wall composition has been examined among cultivars, including across ecotypes, these studies have focused on a single stage (Lemus et al., 2002; Hu et al., 2010).

This work reports the cell wall composition, ED, and lignin biosynthesis GE of a series of developmentally matched samples from A4, AP13, and VS16 switchgrass genotypes. We find that the measured cell wall parameters, especially phenylpropanoid content, vary across development and in many cases among genotypes. For example, VS16 is more digestible than A4 and AP13 at earlier developmental stages but not at reproduction. Generally, expression of phenylpropanoid biosynthesis genes precedes lignin accumulation, suggesting that early GE may be an indicator of cell wall properties later in development.

## Materials and Methods

### Plant Material

Switchgrass (*Panicum virgatum* L.) genotypes A4, AP13, and VS16 were grown in a greenhouse under approximately a 14-h day/10-h night photoperiod at 20–30°C in Norman, Oklahoma. Plants were watered once a week with deionized water and fertilized every 3 months with ∼15-g Osmocote 19-6-12 (Scotts). Single ramets from each genotype were propagated in 5-L pots containing a soil mixture of peat and topsoil (1:1) until producing multiple tillers. A total of nine plants, three biological replicate clones of each genotype, were grown. Harvesting took place between April and July.

### Sample Collection

**FIGURE 1:**
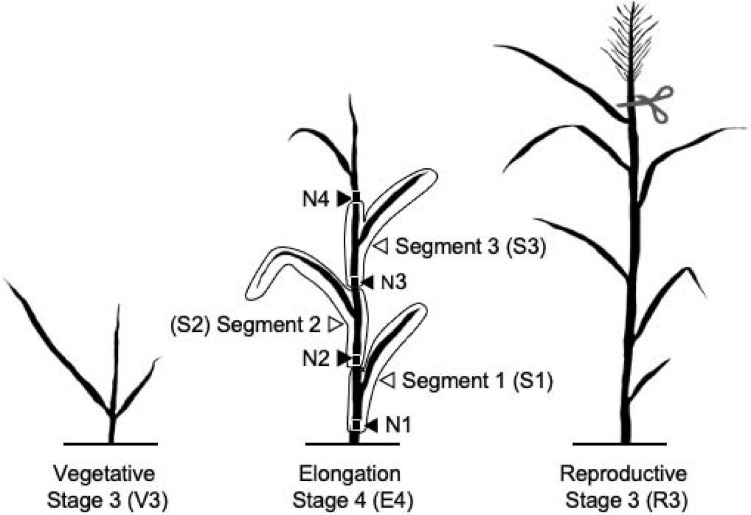
Diagram of switchgrass stages and segments characterized in this experiment. N indicates nodes. Panicle was removed from the R3 tillers.

Switchgrass plant developmental stages were determined as previously described (Moore et al., 1991). Single tillers for the vegetative 3 (V3), elongation 4 (E4), and reproductive 3 (R3) stages were collected from each biological replicate. All harvesting took place between 9 and 11 a.m. local time. Each tiller was harvested by cutting the tiller 3–4 cm above soil level, just beneath the first node, N1 (Figure 1). The sample was immediately chopped into small pieces with heavy-duty scissors into 50-ml conical tubes, followed by quick-freezing in liquid nitrogen. For each plant, an additional E4 tiller was further dissected into three segments. These consisted of S1 (lower, more mature) including the stem from just below the first node 1 (N1) above the soil to just below the second node and including the entire bottom leaf sheath and blade; S2 (middle, intermediate maturity) including the stem just below the second node to the stem just below the third internode and the associated leaf; and S3 (upper, least mature) including the stem just below the third node to the stem just below the fourth internode and the associated leaf uppermost node 4. Node 4 and all distal leaf and stem material were discarded. Samples were homogenized into fine powder in liquid nitrogen using mortars and pestles and stored at −70°C.

### Alcohol Insoluble Residue Preparation

Alcohol insoluble cell wall residue (AIR) preparation and subsequent cell wall analysis were carried out essentially as described (Bartley et al., 2013a). Briefly, ∼100 mg of fresh frozen, ground tissue was treated 4–5 times with a 95% (v/v) ethanol wash for 30 min at 100°C in a thermomixer and centrifugation at 10,000 *g* for 10 min, with the supernatant removed after each wash. AIR was further washed three times with 70% (v/v) ethanol and then vacuum dried. The AIR was destarched using amylase (0.3 U/10 mg AIR) followed by amyloglucosidase (0.33 U/10 mg AIR) and pullulanase (0.04 U/10 mg AIR).

### Carbohydrate Composition

Cell wall carbohydrates were measured as previously described (Bartley et al., 2013a). Briefly, 2-mg destarched AIR was treated with 2 M of trifluoroacetic acid (TFA) at 120°C for 1 h. Monosaccharides were quantified relative to standards using high-performance anion exchange chromatography (HPAEC) with pulsed amperometric detection on an Dionex ICS-3000 system equipped with an electrochemical detector and a 4 × 250 mm CarboPac PA20 column, following the method described previously. The remaining glucose in the pellet was measured with the anthrone method.

### Lignin Content

Acetyl bromide soluble lignin content was used as a proxy for lignin content (Fukushima and Hatfield, 2004). For this analysis, 2 mg of destarched AIR was treated with 100 μl of freshly made acetyl bromide solution (25% v/v acetyl bromide in glacial acetic acid) for 3 h in a screw-cap tube at 50°C with continuous 1,050-rpm shaking and occasional gentle vortexing every 15 min for the last hour. Quantification in a 96-well plate and lignin content estimation were as described (Bartley et al., 2013a).

### Hydroxycinnamic Acid Content

Destarched AIR (3 mg) was saponified using 500 μl of 2 M of NaOH with continuous mixing at 300 rpm in a thermomixer at 25°C for 24 h. The solution was then neutralized with 100 μl of concentrated hydrochloric acid (HCl) and extracted with 300 μl of ethyl acetate. Samples were dried and dissolved in 50% (v/v) methanol for HCA analysis using a Dionex Ultimate 3,000 high-performance liquid chromatography (HPLC) system. Details for the HPLC protocol and run parameters were as previously described, except that detection was at 305 nm (Bartley et al., 2013a).

### Enzymatic Digestibility

ED pretreatment conditions were analyzed using a high-throughput assay system, with minor changes, as previously described (Santoro et al., 2010). Specifically, 2 mg of R3 greenhouse-grown ground total biomass from A4 and AP13 was pretreated with 150 μl of aqueous solution at different NaOH concentrations (0, 0.62, 1.5, 3.0, 6.2, 9.4, 12.5, 15.0, 30, and 62 mM) for 1 h at 90°C with continuous shaking. After cooling and adjusting the pH to 4.8, we carried out ED for 20 h at 50°C with a mixture of enzymes (Accellerase 1,000, Genencor, Rochester, NY) in 30 mM of citrate buffer (pH 4.5) plus 0.01% sodium azide.

For ED assays with developmental samples, destarched AIR (2 mg) was suspended in 100 μl of 100 mM citrate buffer (pH 5.0) at 30°C for 1 h and then pretreated with 1.5 mM NaOH at 100°C for 1 h with continuous shaking. After being cooled to room temperature and neutralized, the slurry was incubated with a 1:1:1 cocktail of the Novozymes enzymes cellulose (NS50013, diluted 1:10), β-glucosidase (NS50010, diluted 1:100), and xylanase and other minor enzymes (NS22002, diluted 1:10) at 50°C with continuous shaking. The released reducing sugars were quantified by 3,5-dinitrosalicylic (DNS) assay as reported (Bartley et al., 2013a).

### Identification and Analysis of Caffeic Acid 3-*O*-Methyltransferase Sequences

Caffeic acid 3-*O*-methyltransferase (COMT) locus IDs and sequences were obtained from the literature and through BLASTP searches of genome databases. Sequences were screened for the presence of the *O*-methyltransferase domain (PF0089.18) using HMMER with the online search tool (Potter et al., 2018). Sequences lacking this domain were excluded. We obtained *Arabidopsis* COMT (AtCOMT1, *AT5G54160*) from TAIR and related sequence from the NCBI GenBank (NP_200227). We identified seven *COMT* sequences from the *P. virgatum* v4.1 draft AP13 genome sequence available through Phytozome (DOE-JGI^1^; locus ID, protein name: *Pavir.6NG060500.1.p*, PvCOMT1a; *Pavir.6KG070300.1.p*, PvCOMT1b; *Pavir.2NG564200.1.p*, PvCOMT2a; *Pavir.2KG513400.1.p*, PvCOMT2b; *Pavir.2NG567300.1.p*, PvCOMT2c; *Pavir.1KG549300.1.p*, PvCOMT3a; and *Pavir.1NG551300.1.p*, PvCOMT3b). PvCOMT1 and PvCOMT2 were named and characterized previously (Wu et al., 2019); however, COMT1b and COMT2c lack PF0089.18 in this version of the annotation, and we excluded them from further analysis. Other COMT protein sequences were found with an orthologous groups search in Phytozome using *AtCOMT*, in the NCBI GenBank database (Benson et al., 2011), and from the literature on genetically and biochemically characterized COMTs. These are as follows: for *Amborella trichopoda* (*AmTr_v1.0_scaffold00001.509*, AmTrCOMT1), for *Arabidopsis* (*AT3G5310*, AtCOMTlike11), for *Brachypodium distachyon* (*Bradi1g14870*, BdCOMT1; *Bradi3g16530*, BdCOMT4, or BdCOMT6; *Bradi2g02380*, BdCOMT2; *Bradi2g02390*, BdCOMT3) (Wu et al., 2013), for safflower (*Carthamus tinctorius*: BAG71895, CtCAldOMT1), for barley (*Hordeum vulgare*: *HORVU7Hr1G082280.1*, HvCOMT1; *HORVU3Hr1G116770.1*, HvCOMT2; *HORVU6Hr1G000040.1*, HvCOMT3) (Daly et al., 2019), for alfalfa (*Medicago sativa*: AAB46623, MsCOMT1) (Zubieta et al., 2002), for tobacco (*Nicotiana tabacum*: X74452.1, NtCOMT1; X71430.1, NtCOMT2) (Maury et al., 1999), for rice (*Oryza sativa*: *LOC_Os08g06100.1*, OsCAldOMT1 or OsCOMT1; *LOC_Os02g57760*, OsCOMT2) (Koshiba et al., 2013), for poplar (*Populus trichocarpa*: ACC63886.1, PtCALdOMT1) (Wang et al., 2015), for *Setaria italica* (*Seita.6G055900.1*, SiCOMT1); for *Sorghum bicolor* (*Sorbic.007G047300.1.p*, SbCOMT1 or BMR12; *Sorbic.004G351400.1.p*, SbCOMT2) (Green et al., 2014), and for maize (*Zea mays*: *GRMZM5G814904_P01*, ZmCOMT or ZmBMR3; *GRMZM2GO82007*, ZmCOMT2).

For each COMT sequence, the *O*-methyltransferase domain was identified using a MOTIF search (KEGG, 2014). Multiple sequence alignment of the *O*-methyltransferase domains was performed in ClustalW (Thompson et al., 1994). Phylogenetic reconstruction was conducted using the maximum likelihood model in MEGA X with the LG amino acid substitution matrix and a discrete Gamma distribution (parameter = 1.1485) with five rate categories and 1,000 bootstrap replications (Kumar et al., 2018).

### RNA Isolation and Quantitative Reverse Transcription-PCR

Primers for quantitative reverse transcription-PCR (qRT-PCR) of phenylpropanoid pathway genes were obtained from the literature (Shen et al., 2012, 2013). Primers for *COMT* gene family members were designed using default parameters of Primer Express software (version 3.0) (Applied Biosystems, Foster City, CA, United States) and selected based on low conservation among homologs. Sequences are listed in Supplementary Table 1.

Total RNA was isolated from 100 mg of frozen ground tissue using the RNeasy Plant Mini Kit (Qiagen, Germantown, MD, United States) following manufacturer’s instructions. RNA integrity was checked on a 1% (w/v) agarose gel and from the 260- to 280-nm absorbance ratio determined with a Take3 plate on a SynergyHT reader (BioTek, Winooski, VT, United States). For first-strand cDNA synthesis, 1 μg of total RNA was subjected to Turbo-DNase (Ambion, Austin, TX, United States) treatment as described in the manufacturer’s protocol followed by cDNA synthesis using the primer d(T)_2__0_VN primer (Sigma, St. Louis, MO, United States) and SuperScript III reverse transcriptase (Life Technologies, Grand Island, NY, United States). Reactions were performed as described previously (Lin et al., 2016) using a Bio-Rad CFX96 thermocycler (Bio-Rad, Hercules, CA, United States) in a white 96-well plate with optical sealing film (Bio-Rad) in 10-μl final volume with 1 μM each of gene specific primers, 2 μl of cDNA, and 5 μl of 2 × SsoFAST EvaGreen Mastermix (Bio-Rad). The following protocol was used for all qPCR reactions: 95°C for 30 s, 40 cycles of 95°C for 1 s, and 60°C for 5 s, followed by default dissociation curves conducted by heating from 60 to 95°C to ensure a single target amplicon. LinRegPCR software was used to estimate PCR efficiency (Ramakers et al., 2003), and relative expression of each gene was determined from efficiency-adjusted ΔCq values. Microsoft Excel was used to calculate means and standard errors of three biological replicates, each measured with three technical replicates.

### Data Analysis and Statistics

Analysis of variance (ANOVA), Tukey’s range test, correlation analyses, and principal component analysis (PCA) were carried out in R (version 2.15.2) (R Development Core Team, 2010). ANOVA was used to detect significant differences of cell wall composition and morphological characteristics among the three genotypes, developmental growth stages, and tiller segments. A Tukey’s range test was performed after ANOVA to detect significant differences at the *P* < 0.05 level, which are indicated by different letters in the figures and tables. The R package “stats” with the function “princomp” was used for PCAs.

Gini correlation analysis was employed to identify relationships among cell wall traits and between cell wall traits and GE. Gini correlation analysis is a hybrid parametric and non-parametric correlation method that uses both rank and value information (Ma and Wang, 2012). It is more tolerant to outliers, less dependent on sample size and data distribution, and better at detecting non-linear relationships than other correlation methods (Ma and Wang, 2012). Gini correlation coefficients (GCCs) and corresponding *P*-values were calculated using the “rsgcc” R package with 2,000 permutation tests (Ma and Wang, 2012). False discovery rate *q*-values were derived with the “*q*-value” R package (Storey, 2002; Storey and Tibshirani, 2003). For the correlations among cell wall parameters, we compared the GCC results with Spearman correlation coefficients (SCC) and Pearson correlation coefficients (PCCs) produced with the “psych” R package (Supplementary Table 2; Revelle, 2018). Most significant correlations were detected by all three methods, with similar coefficients, *P*, and *q*-values. PCC gave the fewest significant correlations, but all correlations that were significant via GCC were significant either with PCC or with SCC. The correlation network generated using the GCC values in the cell wall parameters dataset with *q* < 0.01 was visualized using Cytoscape version 2.8.3 (Shannon et al., 2003). A significance cutoff of *q* < 0.05 was applied for the correlations between GE and cell wall change values, whereas a cutoff of *q* < 0.01 was used for correlations between GE and cumulative cell wall parameters.

## Results

Switchgrass development has been classified into vegetative (V), elongation (E), and reproductive (R) stages (Moore et al., 1991; Hardin et al., 2013). At each stage, different segments (phytomers) develop acropetally, with the upper internodes developing after the bottom segments. To address how changes in development among genotypes alter cell wall properties, we analyzed lignin, hydroxycinnamates, cell wall carbohydrates, cell wall ED, and phenylpropanoid biosynthesis GE of whole tillers at the V3, E4, and R3 stages and three tiller segments at E4 stage, from the bottom segment, S1_E__4_, to the top segment, S3_E__4_ (Figure 1). These samples were studied for three switchgrass genotypes: two lowland, Alamo genotypes (A4 and AP13) and one upland, Summer genotype (VS16). Supplementary Table 3 contains the cell wall and GE data generated in this study.

### Cell Wall Properties

**FIGURE 2:**
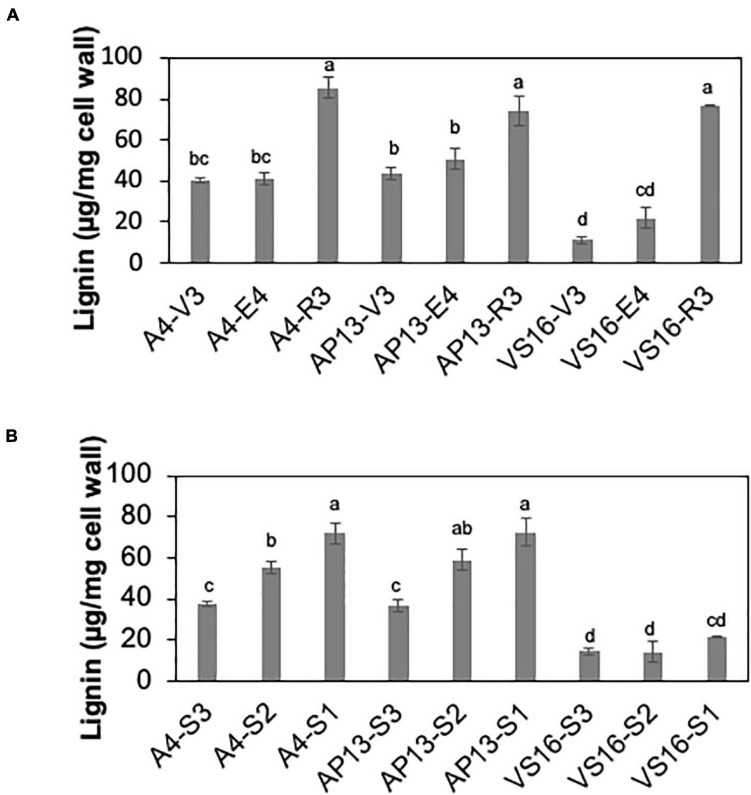
Lignin of switchgrass genotypes A4, AP13, and VS16. Samples are arranged from developmentally young tissues to old tissues. **(A)** Whole tillers at V3, E4, and R3 developmental stages and **(B)** S3, S2, and S1 tissue segments of the E4 tiller as shown in Figure 1. Error bars indicate standard error (*N* = 3). Means with unshared letters are likely to be significantly different [Tukey’s honestly significant difference (HSD), *P* ≤ 0.05].

**FIGURE 3:**
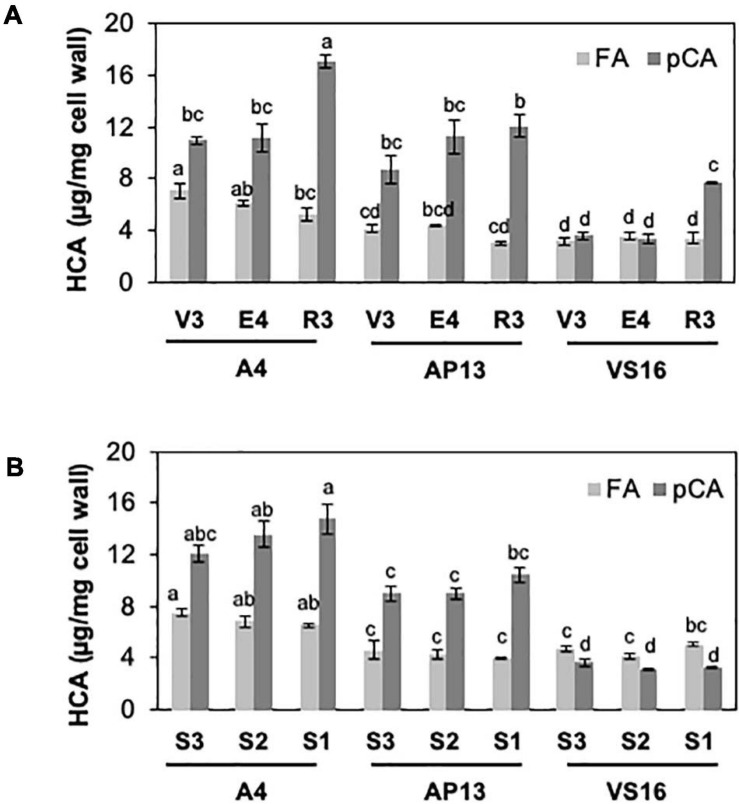
Hydroxycinnamic acids (HCAs), ferulic acid (FA) and *para*-coumaric acid (*p*CA), of switchgrass genotypes A4, AP13, and VS16. Samples, diagramed as in Figure 1, are arranged from young to old stages/segments. **(A)** Whole tillers at V3, E4, and R3 developmental stages and **(B)** S3, S2, and S1 tissue segments of the E4 tiller. Error bars indicate standard error (*n* = 3). Means with unshared letters are likely to be significantly different [Tukey’s honestly significant difference (HSD), *P* ≤ 0.05].

Among measured cell wall components, phenylpropanoids displayed the most significant differences among genotypes and across development. Acetyl bromide soluble lignin significantly varied among growth stages and tiller segments (ANOVA, *P* < 0.05). At the whole tiller level, plants at mature R3 stage contain significantly more lignin than immature V3 and E4 stages in all three genotypes [Figure 2A, Tukey’s honestly significant difference (HSD), *P* < 0.05]. A4 and AP13 have more lignin than VS16 at the V3 and E4 stages but not at the R3 stage. Thus, lignin varies among genotypes in young V3 and E4 stages but converges by the R3 stage. The oldest segment, S1_E__4_, contains significantly more lignin than the younger segment, S3_E__4_ segments of A4 and AP13 but not of VS16 (Figure 2B, Tukey’s HSD, *P* < 0.05), another indication of the lignin development delay in VS16 relative to A4 and AP13. The HCAs, *para*-coumaric acid (*p*CA) and ferulic acid (FA), also differ across genotypes and stages (Figure 3). Consistent with lignin being the major destination for *p*CA in the cell wall, *p*CA tends to increase from young to old plants. The amount of *p*CA at the R3 stage is significantly higher (Tukey’s HSD, *P* < 0.05) than that at V3 and E4 stages in VS16 and A4 (Figure 3A). At each stage, VS16 has less *p*CA than A4 and AP13 and less FA than A4. Across E4 segments, the FA concentration does not vary significantly; in the Alamos, increases in *p*CA with maturity are suggested, but not statistically significant (Figure 3B).

Cellulose and most matrix and pectin monosaccharides are invariant or do not exhibit clear trends across development or among genotypes (Tables 1, 2). Xylose and arabinose decline significantly from the V3 to E4 stage in AP13. TFA-soluble glucose, which derives from mixed linkage glucan and amorphous cellulose, declines with development in AP13 and A4, but not in VS16. Compared with trace amounts of fucose in some other grass species (Lin et al., 2016), switchgrass possesses a higher amount of fucose, ranging from 2.9 to 11 μg/mg, which increases with development. Very few statistically significant differences were detected among segments.

**TABLE 1:** Matrix polysaccharide and non-crystalline cellulose carbohydrate composition (μg/mg) in cell wall residue of across growth stages from A4, AP13, and VS16 switchgrass.

		μg/mg destarched alcohol insoluble residue								
Genotype	Growth stage	Cellulose	Xyl	Glc	Ara	Gal	Fuc	GalA	Rha	GlcA
A4	V3	700 ± 90^a^	140 ± 10^ab^	86 ± 8^ab^	46 ± 3^a^	9.0 ± 1.2^a^	6.5 ± 0.7^abc^	5.5 ± 0.2^ab^	0.6 ± 0.1^b^	0.39 ± 0.06^b^
	E4	600 ± 200^a^	140 ± 10^ab^	120 ± 50^a^	42 ± 5^ab^	7.6 ± 0.4^ab^	5.8 ± 1.6^bc^	5.7 ± 1.1^a^	0.7 ± 0.1^ab^	0.4 ± 0.1^b^
	R3	530 ± 30^a^	130 ± 30^b^	29 ± 8^c^	43 ± 3^ab^	8.6 ± 0.7^ab^	8.4 ± 1.3^ab^	ND	1.1 ± 0.4^ab^	0.50 ± 0.05^b^
AP13	V3	500 ± 100^a^	190 ± 10^a^	43 ± 8^bc^	40 ± 1^ab^	7.7 ± 0.9^ab^	3.0 ± 0.4^c^	3.8 ± 0.4^ab^	0.40 ± 0.06^b^	0.41 ± 0.04^b^
	E4	700 ± 200^a^	150 ± 40^b^	28 ± 8^c^	31 ± 5^bc^	4.9 ± 0.7^cd^	3.2 ± 1.7^bc^	3.3 ± 0.9^ab^	0.5 ± 0.3^b^	0.34 ± 0.09^b^
	R3	600 ± 200^a^	110 ± 20^b^	23 ± 4^c^	34 ± 2^bc^	6.5 ± 0.1^bc^	6.0 ± 0.8^bc^	ND	1.2 ± 0.5^a^	0.43 ± 0.04^b^
VS16	V3	480 ± 40^a^	108 ± 4^b^	37 ± 5^bc^	32 ± 6^bc^	5.8 ± 0.9^bcd^	3.1 ± 0.2^c^	4.9 ± 0.7^ab^	0.3 ± 0.1^b^	0.54 ± 0.02^ab^
	E4	650 ± 20^a^	100 ± 10^b^	24.9 ± 0.5^c^	29 ± 2^c^	4.1 ± 0.3^d^	2.9 ± 0.5^c^	4.0 ± 0.3^ab^	0.31 ± 0.03^b^	0.46 ± 0.06^bc^
	R3	540 ± 40^a^	110 ± 20^b^	39 ± 9^bc^	33 ± 4^bc^	7.5 ± 1.5^ab^	11 ± 4^a^	3 ± 2^b^	0.8 ± 0.1^ab^	0.7 ± 0.2^a^

*Data are mean ± standard deviation. n = 3. In each column, unshared letters indicate significant differences at P ≤ 0.05 as tested by Tukey’s range test.*

*V3, vegetative stage 3; E4, elongation stage 4; R3, reproductive stage 3; Glc, non-crystalline cellulose glucose; Xyl, xylose; Ara, arabinose; Gal, galactose; Fuc, fucose; Rha, rhamnose; GalA, galacturonic acid; and GlcA, glucuronic acid; ND, not detected.*

**TABLE 2:** Carbohydrate composition (μg/mg) of cell wall residue of elongation 4 (E4) tiller segments (S3–S1) from A4, AP13, and VS16 switchgrass.

		μg/mg destarched alcohol insoluble residue								
Genotype	E4 tissue segment	Cellulose	Xyl	Glc	Ara	Gal	Fuc	GalA	Rha	GlcA
A4	S3	600 ± 100^a^	130 ± 40^a^	50 ± 10^a^	38 ± 6^a^	5.6 ± 0.6^ab^	5.2 ± 0.5^bc^	4.2 ± 0.5^a^	0.54 ± 0.02^a^	0.4 ± 0.1^a^
	S2	600 ± 100^a^	141 ± 4^a^	40 ± 7^a^	38 ± 2^a^	5.7 ± 0.4^ab^	4.6 ± 1.0^bc^	4.6 ± 0.6^a^	0.7 ± 0.1^a^	0.43 ± 0.04^a^
	S1	500 ± 100^a^	140 ± 20^a^	50 ± 10^a^	36 ± 1^a^	6.5 ± 0.1^ab^	9.1 ± 1.3^a^	4.2 ± 1.0^a^	1.3 ± 0.3^a^	0.49 ± 0.09^a^
AP13	S3	600 ± 100^a^	100 ± 10^a^	35 ± 2^a^	33 ± 1^a^	5.5 ± 0.6^ab^	3.1 ± 0.9^c^	4.6 ± 1.1^a^	0.5 ± 0.1^a^	0.4 ± 0.1^a^
	S2	500 ± 80^a^	120 ± 20^a^	32 ± 6^a^	32 ± 4^a^	6.6 ± 0.9^ab^	4.4 ± 1.7^bc^	4.3 ± 1.3^a^	0.8 ± 0.3^a^	0.4 ± 0.1^a^
	S1	600 ± 300^a^	130 ± 30^a^	29 ± 5^a^	33 ± 5^a^	7.4 ± 0.9^a^	7 ± 2^ab^	4.9 ± 0.6^a^	1.0 ± 0.3^a^	0.6 ± 0.2^a^
VS16	S3	800 ± 200^a^	110 ± 6^a^	34 ± 6^a^	33 ± 2^a^	5.0 ± 1.3^b^	3.2 ± 0.7^c^	ND	0.5 ± 0.1^a^	0.41 ± 0.09^a^
	S2	500 ± 200^a^	124 ± 3^a^	36 ± 6^a^	35 ± 2^a^	5.7 ± 0.7^ab^	3 ± 2^c^	ND	0.8 ± 0.2^a^	0.5 ± 0.1^a^
	S1	500 ± 10^a^	100 ± 20^a^	32 ± 9^a^	30 ± 5^a^	5.0 ± 1.1^b^	4.0 ± 0.7^bc^	5.6 ± 0.7^a^	1.6 ± 1.6^a^	0.5 ± 0.1^a^

*Data are mean ± standard deviation. n = 3. S1, S2, and S3 are the three tissue segments of E4 stage (see Figure 1), determined according to Moore et al. (1991). In each column, unshared letters indicate significant differences at the P ≤ 0.05 level as tested by Tukey’s range test.*

*Glc, non-crystalline cellulose glucose; Xyl, xylose; Ara, arabinose; Gal, galactose; Fuc, fucose; Rha, rhamnose; GalA, galacturonic acid; GlcA, glucuronic acid; ND, not detected.*

**FIGURE 4:**
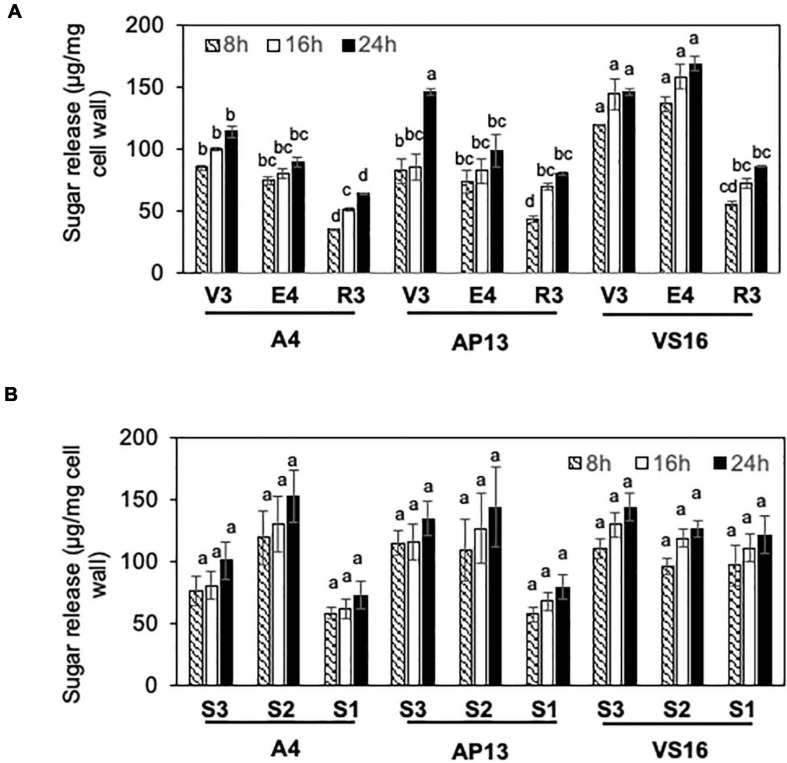
Enzymatic digestibility (ED) of switchgrass genotypes, A4, AP13, and VS16. Samples are diagramed in Figure 1 and are arranged from young to old stages/segments. **(A)** Whole tillers at V3, E4, and R3 developmental stages and **(B)** S3, S2, and S1 tissue segments of the E4 tiller as shown in Figure 1. Cell wall residues from different tissue samples were subjected to enzymatic hydrolysis, and the amount of sugars released after 8, 16, and 24 h of incubation were quantified via DNS assay. Error bars indicate standard error (*n* = 3). Means with unshared letters are likely to be significantly different [Tukey’s honestly significant difference (HSD), *P* ≤ 0.05].

Cell wall residues from the three switchgrass genotypes were further characterized for cellulase ED using pretreatment conditions optimized to show differences among genotypes. To select pretreatment conditions, we evaluated the effect of varying NaOH pretreatment concentrations on yields of lignocellulose-derived sugars by ED of biomass from mature R3 tillers of A4 and AP13. Supplementary Figure 1 shows differences in reducing sugar yields (Δ) between AP13 and A4. At and below 3.0 mM NaOH pretreatment, the ΔAP13-A4 sugar yield was 0.014 mg/g AIR with significant *P*-values (*P* ≤ 0.03); however, at higher NaOH pretreatment concentrations (6.2, 9.4, 12.5, 15, 30, and 62 mM), the difference in sugar yields was not detectable and non-significant (*P* > 0.05). The maximum difference in ED between A4 and AP13 was observed with 1.5 mM NaOH pretreatment at 100°C (Supplementary Figure 1). Under these conditions, differences likely reflect structural properties, such as crosslinking, that prevent enzyme access to cellulose, as opposed to compositional differences, such as variation in the amount of cellulose. For all three genotypes, R3 tillers showed lower ED than V3 and E4 tillers (Figure 4A). Compared with A4 and AP13, VS16 showed significantly higher saccharification efficiency at the V3 and E4 stages (*P* < 0.05). However, as with lignin content, the difference in saccharification efficiency diminished by the R3 stage. Our assay possessed too much noise to statistically distinguish the E4 segments, but the trend was decreased digestibility in the more mature S1_E__4_ segment compared with the others for the Alamo genotypes (Figure 4B).

**FIGURE 5:**
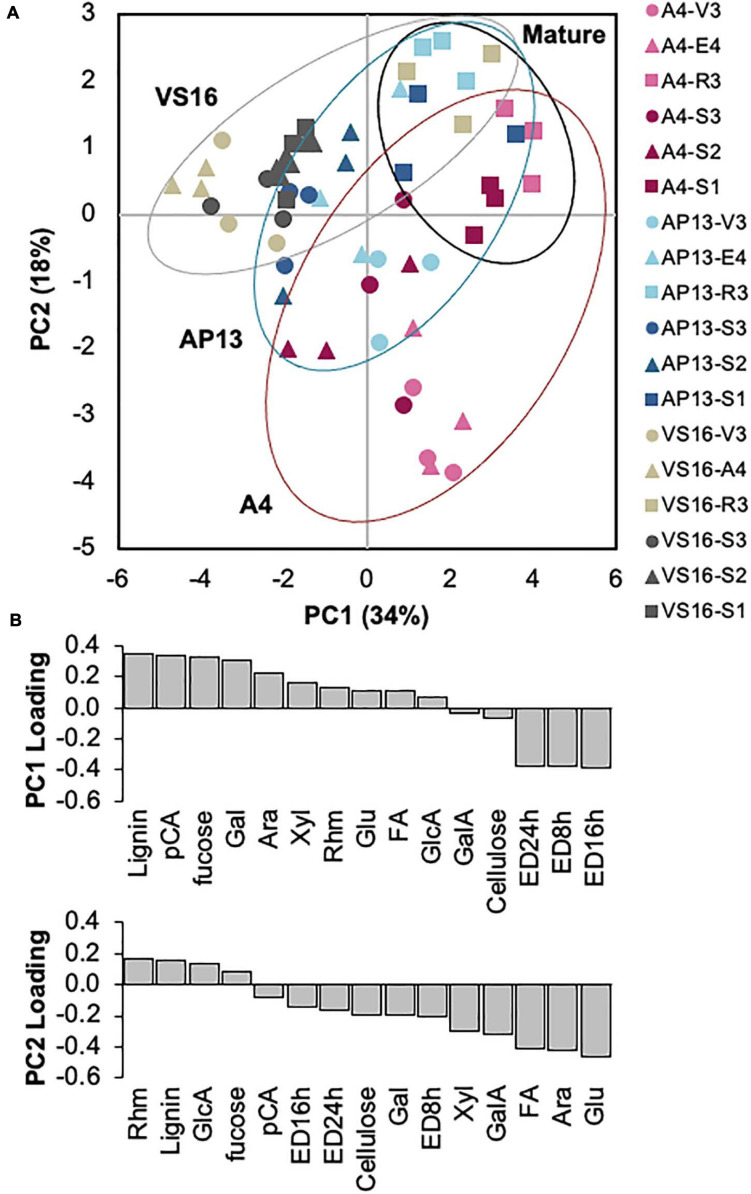
Principal component analysis of cell wall variables indicates segregation by sample age and, partially, by genotype. **(A)** Distribution of samples in the first two principal components. Percentages indicate the variation represented by each component. Red indicates A4; blue, AP13; and gray, VS16. Lighter shading indicates whole tillers, and darker shading indicates segments of the elongation 4 (E4) tiller. Figure 1 diagrams the samples. Circles are younger, triangles are intermediate, and squares are older tillers/segments. **(B)** Loadings of the two major principal components (PC). Ara, arabinose; ED16, ED24, and ED48 h, enzymatic digestibility yield at 16, 24, and 48 h; FA,: erulic acid; Gal, galactose; GalA, galacturonic acid; GlcA, glucuronic acid; Glu, non-crystalline cellulose glucose; *p*CA, *p*-coumaric acid; Rhm, rhamnose.

A PCA of the cell wall parameters indicated that developmentally older samples and the switchgrass genotypes are partially distinguishable (Figure 5A). Principal component 1 (PC1) was negatively influenced by ED but positively influenced by lignin, HCAs, and carbohydrates (Figure 5B). Principal component 2 (PC2) was negatively influenced by ED, HCAs, and carbohydrates including xylose, arabinose, and (non-crystalline cellulose) glucose but positively influenced by lignin and minor monosaccharides. Based on PC1 and PC2 (Figure 5A), A4 and VS16 had divergent cell wall properties, whereas AP13 was intermediate. Plant development also impacts cell wall composition and properties. Mature R3 and S1_*E4*_ samples, shown by squares in Figure 5A, generally have higher values in both principal components than younger samples. As indicated by the spread in the PCA, the cell wall differences among genotypes are larger in younger samples (V3, E4, S3_*E4*_, and S2_*E4*_) than in older samples (R3 and S1).

**FIGURE 6:**
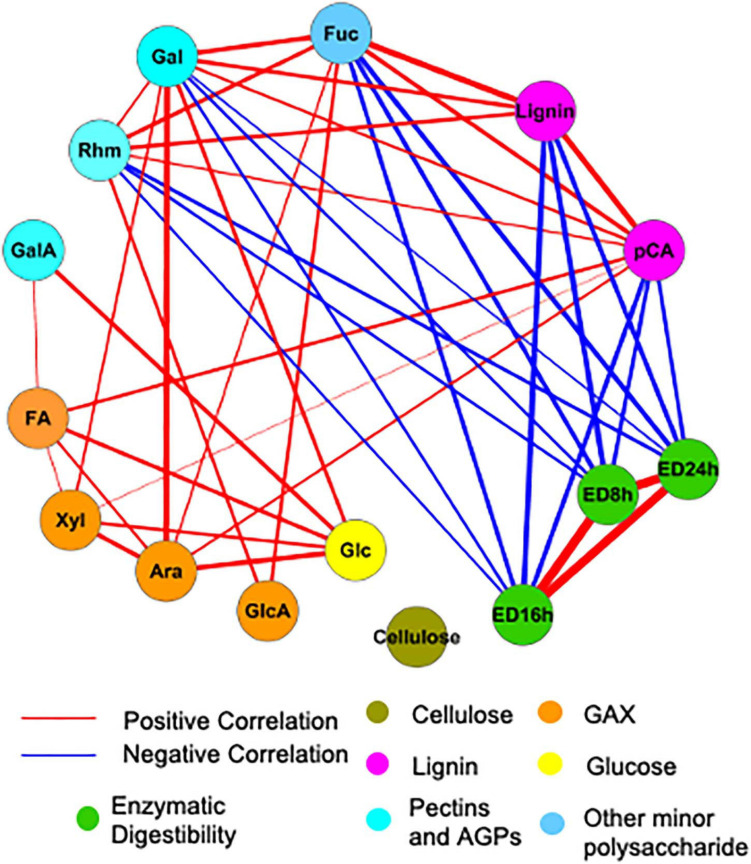
Significant correlations among cell wall components and enzymatic digestibility (ED). Red and blue lines indicate positive and negative correlations, respectively. The thickness of lines is proportional to the absolute value of Gini correlation coefficient. Cell wall components are represented by circular nodes and color-coded based on the cell wall polymers from which they originate. Xyl, xylose; Ara, arabinose; Glc, glucose; Gal, galactose; Rhm, rhamnose; GalA, galacturonic acid; FA, ferulic acid; *p*CA, *p*-coumaric acid; ED, enzymatic digestibility. The glucose is non-crystalline cellulose, trifluoroacetic acid (TFA)-soluble glucose. GAX is glucuronoarabinoxylan. Only correlations with a *q* < 0.01 are shown. Correlation coefficients calculated with Gini, Pearson, and Spearman methods and corresponding *P* and *q*-values are provided in Supplementary Table 2.

To examine the influence of cell wall composition on saccharification efficiency, in particular, we analyzed the correlations among measured cell wall properties using the Gini correlation method (Figure 6). ED at different time points is strongly correlated. As expected, lignin and *p*CA negatively correlate with ED, as do minor sugars such as rhamnose, galactose, and fucose. Other cell wall carbohydrates, like cellulose and arabinoxylan, are not significantly correlated with ED possibly because of their low variation across samples. The correlations among cell wall components in this study are partially consistent with correlations based on a cell wall composition atlas of rice tissues from different stages (Lin et al., 2016). Glucuronoarabinoxylan components, including xylose, arabinose, and glucuronic acid, positively correlate with each other as in rice; however, there are also correlations not observed in rice but observed for switchgrass, like the positive correlation between *p*CA and lignin.

### Phenylpropanoid Biosynthesis Gene Expression

As lignin precursors and HCAs are synthesized by phenylpropanoid biosynthesis genes, we measured expression of selected switchgrass phenylpropanoid biosynthesis genes across the switchgrass genotypes, stages, and internode segments. Transcripts examined correspond to those described in the switchgrass literature, as follows (Shen et al., 2012, 2013): *4-coumarate:CoA ligase* (*4CL*), *coumarate 3’-hydroxylase* (*C3’H*), *cinnamate 4-hydroxylase* (*C4H*), *cinnamyl alcohol dehydeogenase* (*CAD*), *caffeoyl CoA 3-O-methyltransferase* (*CCoAOMT*), *cinnamoyl coenzyme A reductase* (*CCR1*), *ferulate 5-hydroxylase* (*F5H*), and *hydroxycinnamoyl-CoA/shikimate/quinate hydroxycinnamoyltransferase* (*HCT*). Due to the importance of S:G ratios in cell wall recalcitrance, as partially determined by the action of COMT enzymes (Fu et al., 2011), we identified and separately measured transcript abundance from three *COMT* homologs, numbered 1 through 3, with some of our qRT-PCR primers recognizing multiple homeologs in the current version of the switchgrass genome (Supplementary Table 1). Phylogenetic reconstruction of the relationship of the three *COMT* gene product targets relative to COMTs in the literature indicates that PvCOMT1 and PvCOMT2 are orthologous to biochemically and genetically studied barley (Daly et al., 2019), sorghum (Green et al., 2014), rice (Koshiba et al., 2013), and *Brachypodium* (Wu et al., 2013) COMTs, which are also sister to the *Arabidopsis* COMT protein. On the other hand, PvCOMT3 is in a distinct clade that includes OsCOMT2 and is sister to NtCOMT2. In tobacco, *NtCOMT2* transcript was induced in response to virus infection and did not vary with development (Pellegrini et al., 1993).

**FIGURE 7:**
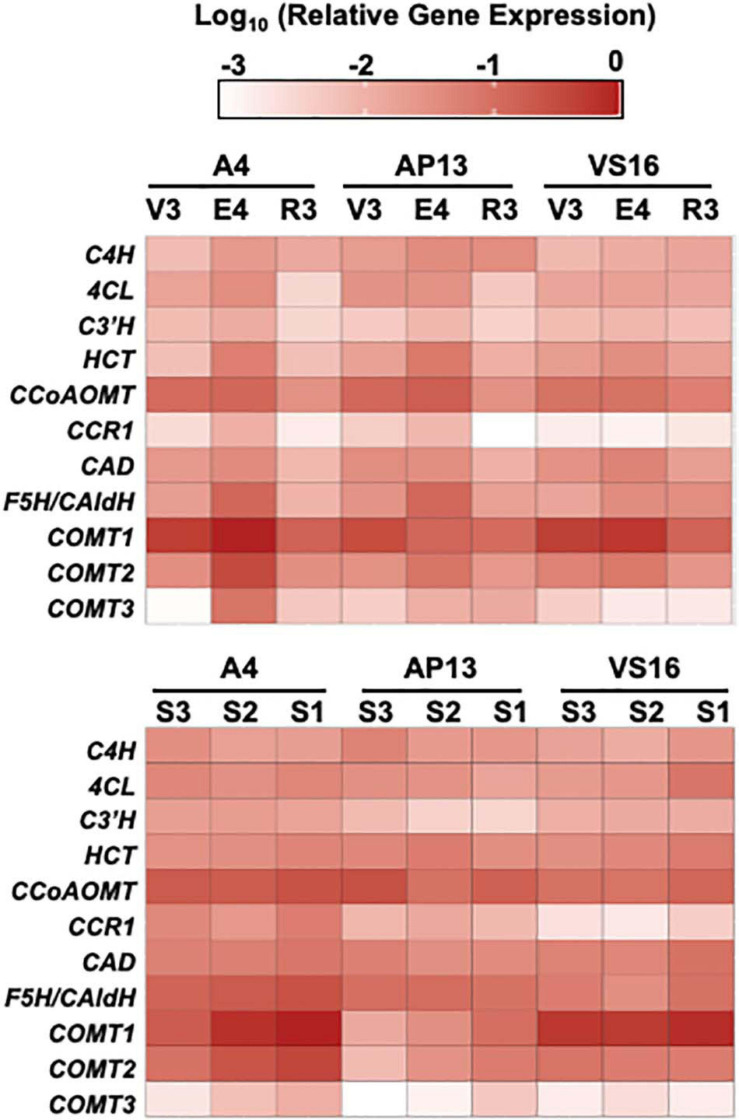
Relative gene expression of phenylpropanoid biosynthesis genes by quantitative RT-PCR. ΔCq values, relative to switchgrass *Ubi10*, were log 10 transformed. Color intensity indicates greater expression. 4CL, 4-coumarate:CoA ligase; C3’H, *para*-coumarate 3’-hydroxylase; C4H, cinnamate 4-hydroxylase; CAD, cinnamyl alcohol dehydrogenase; CCoAOMT, caffeoyl CoA *O*-methyltransferase; CCR, cinnamyl CoA reductase; COMT, caffeic acid 3-*O*-methyltransferase; F5H/Cald5H, ferulate 5-hydroxylase/coniferaldehyde 5-hydroxylase; HCT, *para*-hydroxycinnamoyl-CoA: Quinate/shikimate *p*-hydroxycinnamoyltransferase.

Quantitative real-time PCR measurements for the switchgrass phenylpropanoid biosynthesis genes showed that most genes have similar expression patterns in all three genotypes but vary across development (Figure 7 and Supplementary Table 3). PCA indicates that genotypes, stages, and E4 segments are not distinguished on the basis of the expression of these genes (Supplementary Figure 3), with the lowland Alamos, A4 and AP13, differing more from each other than from the upland VS16. Among the three developmental stages, most genes had the highest expression at the E4 stage, with geometric mean of relative expression being approximately threefold higher than in the R3 and twofold higher than the V3 stage. In the internode segments of A4 and AP13, expression of the *COMT*s increased from the young S3_E__4_ segment to the older S2_E__4_ and S1_E__4_ segments, but this pattern was not clear in VS16. VS16 instead shows an increase with segment maturity for *4CL* and *HCT*. Among the segments across genotypes, the geometric mean across tested genes was the highest in the more mature S1_E__4_ segment, but besides those noted, patterns differed among genes and genotypes.

### Correlations Between Gene Expression and Cell Wall Parameters

To understand how expression of phenylpropanoid biosynthesis GE influences cell wall phenolic compounds and ED (cell wall parameters, CW), we analyzed Gini correlations between these components with various models. The first model (N_GE_ vs. N_CW_) hypothesizes a correlation between concurrent expression of phenylpropanoid biosynthesis genes and cell wall parameters in the same sample (N). For example, this correlates expression in V3 with cell wall features in V3, or expression in S1_E__4_ with cell wall features in the S1_E__4_ segment. Next, in two “delta” models, we compared GE at time N with the change (Δ) in a cell wall component between N and samples later in the developmental series (N_GE_ vs. Δ[(N + 1) - N_CW_]) and (N_GE_ vs. Δ[(N + 2) - N_CW_]). These models tested the hypothesis that expression earlier in development would lead to cell wall properties later in development. For example, the (N_GE_ vs. Δ[(N + 1) - N_CW_]) model includes V3 expression correlations with E4 composition and S1_E__4_ GE with S3_E__4_ CW. Supplementary Table 4 contains the GCC and associated *P* and *q*-values for these N_GE_ vs. N_CW_ and N_GE_ vs. ΔN_CW_ models.

**FIGURE 8:**
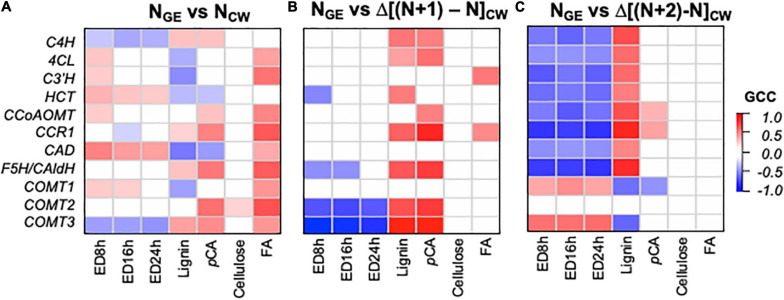
Correlations between phenylpropanoid gene expression (GE) and the change in cell wall (CW) variables between samples reflecting different developmental times indicate that phenylpropanoid biosynthesis gene expression precedes cell wall phenylpropanoid accumulation and a change in enzymatic digestibility (ED) recalcitrance. **(A)** N_GE_ vs. N_CW_ model with gene expression and cell wall composition in the same segment. **(B)** N_GE_ vs. Δ[(N + 1) - N_CW_] model with gene expression in a segment (N) preceding accumulation of phenylpropanoid components. **(C)** N_GE_ vs. Δ[(N + 2) - N_CW_] model with gene expression in a segment long preceding accumulation of phenylpropanoid components. ED#h indicates the enzymatic digestibility yield after # hours of incubation. Color intensity represents magnitude of Gini correlation coefficient (GCC). Only correlations with *q* < 0.05 are shaded. Correlation coefficients, *P*-values, and *q*-values are available in Supplementary Table 4.

Significant correlations (*q* < 0.05) in the concurrent (N_GE_ vs. N_CW_) and Δ delay (N_GE_ vs. Δ[(N + 1) - N_CW_] and N_GE_ vs. Δ[(N + 2) - N_CW_]) models differ qualitatively (Figure 8). In the N_GE_ vs. N_CW_ model (Figure 8A), phenylpropanoid biosynthesis genes show a mix of expected and unexpected correlations. Without exception, correlations between expression and FA, when significant, are positive. With lignin and *p*CA, there are a few expected positive correlations and a few unexpected negative correlations. Similarly, ED, which we expect to be negatively correlated with phenylpropanoid GE, shows mostly positive correlations. In contrast, in the N_GE_ vs. Δ[(N + 1) - N_CW_] model (Figure 8B), a majority of phenylpropanoids synthesis genes, including *C4H*, *4CL*, *CCR1*, *F5H*, and two of the *COMT*s, positively correlate with the change in lignin and *p*CA. A few negative correlations, and no unexpected positive correlations, are significant between GE and ED in this model. Finally, in the model with the larger delay (N_GE_ vs. Δ[(N + 2) - N_CW_], Figure 8C), transcript abundance and the change in lignin abundance are positively correlated for all lignin biosynthesis genes, except the *COMT*s. Likewise, transcript abundance and ED are negatively correlated for all but the *COMT*s. In this model, *p*CA abundance positively correlates with *CCR1* and *CCoAOMT* expression. In summary, the delay models produce more expected significant correlations for lignin, *p*CA, and ED, while the concurrent model shows more expected correlations between GE and FA.

We next asked how phenylpropanoid GE correlates with cumulative cell wall properties in the developmental series, again with various models that include delays and separating genotypes, stages, and segments. We particularly were interested in the question of which samples’ GE indicate cell wall properties at maturity. Table 3 shows the various Gini correlation models tested, sorted based on precision, i.e., the number of expected significant correlations divided by the total significant correlations. The input data are available in Supplementary Table 5. Expected correlations include positive correlations between phenylpropanoid GE and accumulated abundance of phenylpropanoid-derived cell wall components (lignin, *p*CA, and FA) and negative correlations with ED. While unexpected correlations are the opposite, such as significant negative correlations between GE and phenylpropanoid components.

**TABLE 3:** Significant correlations between switchgrass phenylpropanoid biosynthesis gene expression and cell wall properties for various data subsets and relationships, i.e., models.

Model (Gene expression vs. Cell wall properties)^a^	Expected correlations^b^	Unexpected correlations^c^	Precision^d^	Recall^e^
Stages N vs. N + 1	20	0	1.00	0.30
S1_E__4_ vs. R3	14	0	1.00	0.21
S2_E__4_ vs. R3	9	0	1.00	0.14
E4 vs. R3	7	0	1.00	0.11
S3_E__4_ vs. R3	6	0	1.00	0.09
A4 N vs. N + 1	3	0	1.00	0.05
N vs. N	13	5	0.72	0.20
N vs. N + 1	12	6	0.67	0.18
AP13 N vs. N + 1	4	2	0.67	0.06
AP13 N vs. N	2	2	0.50	0.03
VS16 N vs. N + 2	2	4	0.33	0.03
A4 N vs. N + 2	2	5	0.29	0.03
N vs. N + 2	3	12	0.20	0.05
VS16 N vs. N	1	4	0.20	0.02
AP13 N vs. N + 2	1	5	0.17	0.02
Stages N vs. N	1	12	0.08	0.02
A4 N vs. N	0	0	−	0
VS16 N vs. N + 1	0	0	−	0
R3 vs. R3	0	0	−	0
V3 vs. E4	0	0	−	0
V3 vs. R3	0	4	0	0
E4 segments N vs. N	11	0	1.00	0.17
E4 segments N vs. N + 1	10	0	1.00	0.15
E4 segments N vs. N + 2	5	0	1.00	0.08

*Analysis was via Gini correlation with q < 0.01. Correlation coefficients, P-values, and q-values are in Supplementary Table 3.*

*^*a*^Stages refers to whole tillers of the vegetative 3 (V3), elongation 4 (E4), and reproductive 3 (R3). S# refers to the E4 tiller segments. N vs. N is gene expression (GE) and cell wall (CW) properties from the same sample; N vs. N + 1 is GE for the N sample and cumulative CW properties for the immediate subsequent sample, i.e., V3 vs. E4, E4 vs. R3, S1_E__4_ vs. S2_E__4_, and S2_E__4_ vs. S3_E__4_. N vs. N + 2 is GE for the N sample and cumulative CW properties for the more developmentally distant sample, i.e., V3 vs. R3 and S1_E__4_ vs. S3_E__4_. When the switchgrass genotype is given, i.e., AP13, A4, and VS16, only samples for that genotype were analyzed. If unspecified, data from all genotypes were included.*

*^*b*^Expected correlations is the count of positive significant correlations between expression of each phenylpropanoid gene with the cell wall phenylpropanoid-derived components [i.e., lignin, p-coumaric acid (pCA), and ferulic acid (FA)], plus the count of significant negative correlations between expression of each phenylpropanoid gene with enzymatic digestibility (ED) at each of the three time points, 8, 16, and 24 h.*

*^*c*^Unexpected correlations is the count of negative significant correlations between expression of each phenylpropanoid gene with the cell wall phenylpropanoid-derived components (i.e., lignin, pCA, and FA), plus the count of significant positive correlations between expression of each phenylpropanoid gene with ED8, ED16, and ED24.*

*^*d*^Precision in the ratio of expected correlations to all observed correlations, i.e., true positives/(true positives + false positives).*

*^*e*^Recall is the ratio of observed expected correlations to all 66 expected correlations, i.e., true positives/(true positives + false negatives).*

Though limited by the small number of samples, genotypes, and transcripts examined, our result is that cumulative CW properties are generally highly correlated with GE at a recent preceding series sample. For example, the highest performing model was for the N_GE_ vs. (N + 1)_CW_ for developmental stages (excluding the segment data). This data subset revealed 20 expected correlations and no unexpected correlations (*q* < 0.01, Table 3 and Supplementary Figure 4). The next four models with high numbers of expected correlations and no unexpected correlations are for E4_G__E_ to R3_C__W_ and for E4_G__E_ segments (S1, S2, and S3) to R3_C__W_ (Table 3). In these models, expression of *CCR*, the *COMT*s, and *F5H* in the entire E4 tiller or in E4 segments shows significant correlations with lignin or ED in R3 samples (Supplementary Figure 4). In contrast to the delta CW analysis in Figure 8, the models that return the fewest expected correlations are those that correlate expression in V3 with later composition, e.g., V3_G__E_ vs. R3_C__W_.

## Discussion

This study examined the generality of switchgrass cell wall synthesis GE and cell wall composition among genotypes toward establishing methods for analyzing the determinants of cell wall composition and biorefining suitability across genotypes. Among the examined switchgrass genotypes at the V3 and E4 stages, lignin content, HCAs, and cell wall digestibility varied significantly, but the differences diminished at the R3 stage. The low abundance of lignin and HCAs in upland VS16 samples relative to lowland A4 and AP13 samples was associated with high ED. Due to superior digestibility, VS16 biomass at V3 and E4 stages has the potential to yield more biofuel than A4 and AP13, but VS16 loses this advantage by the R3 stage. A caveat of this work is that the greenhouse conditions might have had a greater influence on upland, VS16 (Casler et al., 2004). Still, the combined effect of developmental stage and genetic background should be considered when selecting cultivars with promising cell wall traits or when genetically modifying plant biomass for higher saccharification efficiency (Ashworth et al., 2017). For example, a plant with delayed lignin accumulation, such as that exhibited by VS16 under the tested conditions, may have a greater time to accumulate biomass before lignin deposition decreases saccharification efficiency.

In contrast to phenylpropanoids, we observed fewer and less pronounced differences in sugar composition among genotypes and stages. While lignification and hydroxycinnamates are determining factors for reduced saccharification and cell wall digestibility (Dien et al., 2006; McCann and Carpita, 2008; Buanafina, 2009), non-cellulosic polysaccharide components from xylan, pectins, arabinogalactan proteins, and xyloglucan also reduce digestibility (DeMartini et al., 2013; Chung et al., 2014; Li et al., 2019). Indeed, the negative correlations that we observe between ED and the pectic sugars, rhamnose and galactose, are consistent with these observations. Still, in our results, polysaccharide component abundance changed little across developmental stages and tiller segments. Thus, lignin remains a key genetic engineering target since it varies across development and among genotypes, while polysaccharide content appears relatively static and perhaps less tolerant of manipulation.

The relative timing of phenylpropanoid biosynthesis GE and cell wall properties provides targets for further study of cell wall-related GE strategies to control harvested biomass digestibility. Phenylpropanoid biosynthesis GE is the highest at the E4 stage, apparently leading to the approximately twofold increase in lignin from the E4 to R3 stage. This result was consistent with our overall analysis indicating that GE in prior stages (N_GE_ vs. Δ[(N + 1) - N_CW_] and N_GE_ vs. Δ[(N + 2) - N_CW_] models) rather than concurrent expression (N_GE_ vs. N_CW_ model) captures expected positive correlations between phenylpropanoid biosynthesis transcript and a change in lignin abundance (Figure 8). Indeed, lignin deposition may be a slow process, in that it includes precursor synthesis, dehydrogenation, and polymerization (Raes et al., 2003). An additional explanation consistent with the data is that lignin undergoes turnover, i.e., breakdown and recycling, that is more rapid than accumulation during earlier developmental stages. Besides evidence for changes to the lignin-containing Casparian strip of roots (Vermeer et al., 2014), we are unable to find clear evidence of this in the literature, suggesting that additional research is required. Consistent with our results, transcriptomics of maize internode subsegments also showed that phenylpropanoid biosynthesis peaks prior to maximal lignin accumulation (Zhang et al., 2014). In our analysis, which includes different genotypes, the observation that correlations between GE and a decrease in ED are the most negative in the N_GE_ vs. Δ[(N + 2) - N_CW_ model hints that high phenylpropanoid biosynthesis GE earlier in development may establish later digestibility recalcitrance. Furthermore, the delay models suggest that expression of all the examined phenylpropanoid biosynthesis genes correlates with lignin accumulation, albeit with different relationships with different genes. Potentially indicating an absence of functional conservation across species, this includes *COMT3*, for with the tobacco ortholog did not vary with development (Pellegrini et al., 1993).

The HCAs show different accumulation kinetics and GE correlations compared with lignin. Though FA is also synthesized by phenylpropanoid biosynthesis genes, it is mainly esterified to arabinoxylan (Bartley et al., 2013a). This different polymer destination may explain why FA positively correlates with phenylpropanoid biosynthesis genes in the concurrent (N_GE_ vs. N_CW_) model but not in the delay models. It may also indicate active turnover of FA and/or xylan in the wall (Franková and Fry, 2011). These patterns—early expression controlling lignin accumulation, but sustained expression controlling FA accumulation—may be used to insinuate regulators and other enzymes involved in these alternative processes. Likewise, the difference in correlations between phenylpropanoid biosynthesis GE with *p*CA and lignin abundance (Figure 8) hints at extra control of *p*CA synthesis, which is consistent with new evidence that hydroxycinnamoylated monolignols are synthesized by different enzymes than un-acylated monolignols (Takeda et al., 2018). This notion is supported by the absence of a correlation between *C3’H* and *p*CA abundance, since reduction of *C3’H* in rice does not alter abundance of cell wall monolignols esterified with *p*CA (Takeda et al., 2018).

## Conclusion

In conclusion, this study advances our knowledge of the relationships between switchgrass developmental stage and genetic background with GE, cell wall composition, and digestibility. Under greenhouse growth conditions, lignin, HCAs, and ED differ significantly, and in a developmentally dependent manner, among genotypes. Though developmental cell wall profiles differed among genotypes, general relationships emerged. Correlations between cell wall phenolics and phenylpropanoid biosynthesis GE revealed that FA accumulation may precede, or be less stable than, *p*CA and lignin accumulation. Expression of phenylpropanoid biosynthesis genes in mid-development seems likely to be an indicator of cell wall properties, especially lignin, *p*CA, and ED, at harvest. Systems analysis with more genotypes and global expression analysis will be needed to confirm these conclusions. The recommendation from the analysis presented here would be that such studies should probe GE in whole or partial tillers at mid-development as an indicator of biomass properties at harvest.

## Data Availability Statement

The datasets presented in this study can be found in online repositories. The names of the repository/repositories and accession number(s) can be found in the article/Supplementary Material.

## Author Contributions

PS, FL, and LB conceived and designed the study and wrote the manuscript. PS and ST generated the data. All authors analyzed the data, edited the manuscript, and approved the final version.

## Conflict of Interest

The authors declare that the research was conducted in the absence of any commercial or financial relationships that could be construed as a potential conflict of interest.

## Publisher’s Note

All claims expressed in this article are solely those of the authors and do not necessarily represent those of their affiliated organizations, or those of the publisher, the editors and the reviewers. Any product that may be evaluated in this article, or claim that may be made by its manufacturer, is not guaranteed or endorsed by the publisher.

## Footnotes

1
https://phytozome-next.jgi.doe.gov

## Supplementary Material

Supplementary Figure 1 Dependence of enzymatic deconstruction results on NaOH concentrations.

Supplementary Figure 2 Caffeic acid 3-O-methyltransferase (COMT) phylogeny.

Supplementary Figure 3 Principle component analysis of phenylpropanoid biosynthesis gene expression data.

Supplementary Figure 4 Heatmaps of Gini correlations between phenylpropanoid biosynthesis gene expression and cell wall properties for highest performing models.

Supplementary Table 1 qRT-PCR Primers.

Supplementary Table 2 Significant correlations among cell wall components.

Supplementary Table 3 Cell wall and gene expression data.

Supplementary Table 4 Gini correlation results between phenylpropanoid gene expression and cell wall traits (N vs. N), or a change in cell wall traits (N vs. N +1 and N vs. N +2).

Supplementary Table 5 Gini correlations between phenylpropanoid gene expression and cell wall parameters for various models and data subsets.

Supplementary Data 1 Caffeic acid 3-O-methyltransferase (COMT) domain sequences.
