# Supplementary material for: Phenylpropanoid Biosynthesis Gene Expression Precedes Lignin Accumulation During Shoot Development in Lowland and Upland Switchgrass Genotypes
Source: Front Plant Sci. 2021 Aug 9;12:640930. doi: 10.3389/fpls.2021.640930 (PMC8380989; doi:10.3389/fpls.2021.640930)
Supplement: Supplementary Figure 1 — Dependence of enzymatic deconstruction results on NaOH concentrations. [file Data_Sheet_1.docx]

**Phenylpropanoid Biosynthesis Gene Expression Precedes Lignin Accumulation during Shoot Development in Lowland and Upland Switchgrass Genotypes**

Prasenjit Saha, Fan Lin, Sandra Thibivilliers, Nicolas Santoro, Laura E. Bartley

Supplementary Material

**Table S1**. qRT-PCR Primers.

**Table S2.** Cell wall and gene expression data. (Excel)

**Table S3.** Gini correlation results for gene expression versus cell wall composition. (Excel)

**Figure S1**. Dependence of enzymatic deconstruction results on NaOH concentrations.

**Figure S2.** Caffeic acid 3-O-methyltransferase (COMT) phylogeny.

**Figure S3.** Principle component analysis of phenylpropanoid biosynthesis gene expression data.

Figure S4. Heatmaps of Gini Correlations between phenylpropanoid biosynthesis gene expression and cell wall properties for highest performing models.

**Data S1.** Caffeic acid 3-O-methyltransferase (COMT) domain sequences

**Supplementary Table 1**. Primer sequences used in this study.

| Primer name | Sequence (5´🡪 3´) | Switchgrass v 4.1 Locus ID | Reference |
| --- | --- | --- | --- |
| *PvUBQ10-qFn* | TTCGTGGTGGCCAGTAAGC |  | Shen H et al. 2013^a^ |
| *PvUBQ10-qRn* | AGAGACCAGAAGACCCAGGTACAG |  | Shen H et al. 2013 |
| *Pv4CL1_1179F* | CGAGCAGATCATGAAAGGTTACC |  | Shen H et al. 2012^b^ |
| *Pv4CL1_1251R* | CAGCCAGCCGTCCTTGTC |  | Shen H et al. 2012 |
| *PvF5H_1720F* | CTCTTCTATTTGTGCGTGTAACTGTGT |  | Shen H et al. 2012 |
| *PvF5H_1792R* | CAGCCCTATAGCATCGACATGA |  | Shen H et al. 2012 |
| *PvC3H1_739F* | TTGAGATGGTTGTGTCCGCTTA |  | Shen H et al. 2012 |
| *PvC3H1_803R* | AGGCGGTCCCTTCTCTCATT |  | Shen H et al. 2012 |
| *PvCCR1_112F* | GCGTCGTGGCTCGTCAA |  | Shen H et al. 2012 |
| *PvCCR1_187R* | TCGGGTCATCTGGGTTCCT |  | Shen H et al. 2012 |
| *PvC4H1_1534F* | GGGCAGTTCAGCAACCAGAT |  | Shen H et al. 2012 |
| *PvC4H1_1611R* | CGCGTTTCCGGGACTCTAG |  | Shen H et al. 2012 |
| *PvCAD_116F* | TCACATCAAGCATCCACCATCT |  | Shen H et al. 2012 |
| *PvCAD_184R* | GTTCTCGTGTCCGAGGTGTGT |  | Shen H et al. 2012 |
| *PvHCT_973F* | GCAGAAGGAGCAGCAGTCATC |  | Shen H et al. 2012 |
| *PvHCT_1035R* | CGAGCGGCAATAGTCGTTGT |  | Shen H et al. 2012 |
| *PvCCOMT_966F* | CCGTCTTTCTTTTTTTTGGCTCTT |  | Shen H et al. 2012 |
| *PvCCOMT_1029R* | GCATGAAAATGATGACAGTTTCCA |  | Shen H et al. 2012 |
| *PvCOMT1_qF* | AGATCTGTTGTCGCGCATGTGCT | *Pavir.6KG070300* | This study |
| *PvCOMT1_qR* | GGAATCCAGAACAAGGATCGATGG |  | This study |
| *PvCOMT2_qF* | ACACATGGGCCATTGAGTTCAC | *Pavir.2NG564200, Pavir.2KG513400* | This study |
| *PvCOMT2_qR* | TGACGAAGACCCATTCATCGAA |  | This study |
| *PvCOMT3_qF* | TCTACCTCGACCCATTCTAT GCCA | *Pavir.1KG549300* | This study |
| *PvCOMT3_qR* | GCTGTTCGACTCAACTGTCACAAG |  | This study |

^a^ Shen H, Mazarei M, Hisano H, Escamilla-Trevino L, Fu C, Pu Y, Rudis MR, Tang Y, Xiao X, Jackson L *et al*: **A genomics approach to deciphering lignin biosynthesis in switchgrass**. *The Plant Cell* 2013, **25**(11):4342-4361.

^b^ Shen H, He X, Poovaiah CR, Wuddineh WA, Ma J, Mann DG, Wang H, Jackson L, Tang Y, Stewart CN, Jr. *et al*: **Functional characterization of the switchgrass (Panicum virgatum) R2R3-MYB transcription factor PvMYB4 for improvement of lignocellulosic feedstocks**. *New Phytologist* 2012, **193**(1):121-136.


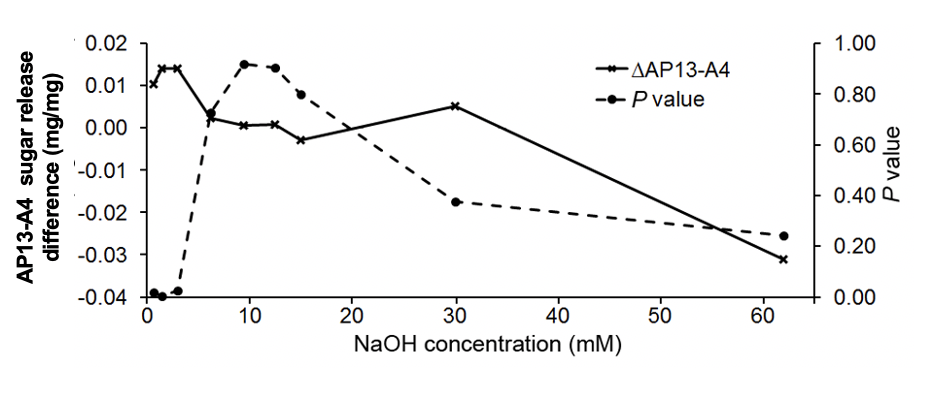


**Figure S1.** Differences in enzymatic digestibility between reproductive stage AP13 and A4 genotypes is only significant at low [NaOH] pretreatment concentrations (90°C, 1 h). The difference of enzymatic deconstruction yield of AP13 and A4 is indicated by the solid line, and the corresponding Student’s t-test *p*-*values* are indicated by the dash line. At low NaOH, AP13 gives 10% greater enzymatic digestibility yields than A4. N = 3 bioreplicates. Enzymatic digestion was for 20 h. Each point was measured with technical triplicates that were combined prior to statistical analysis.


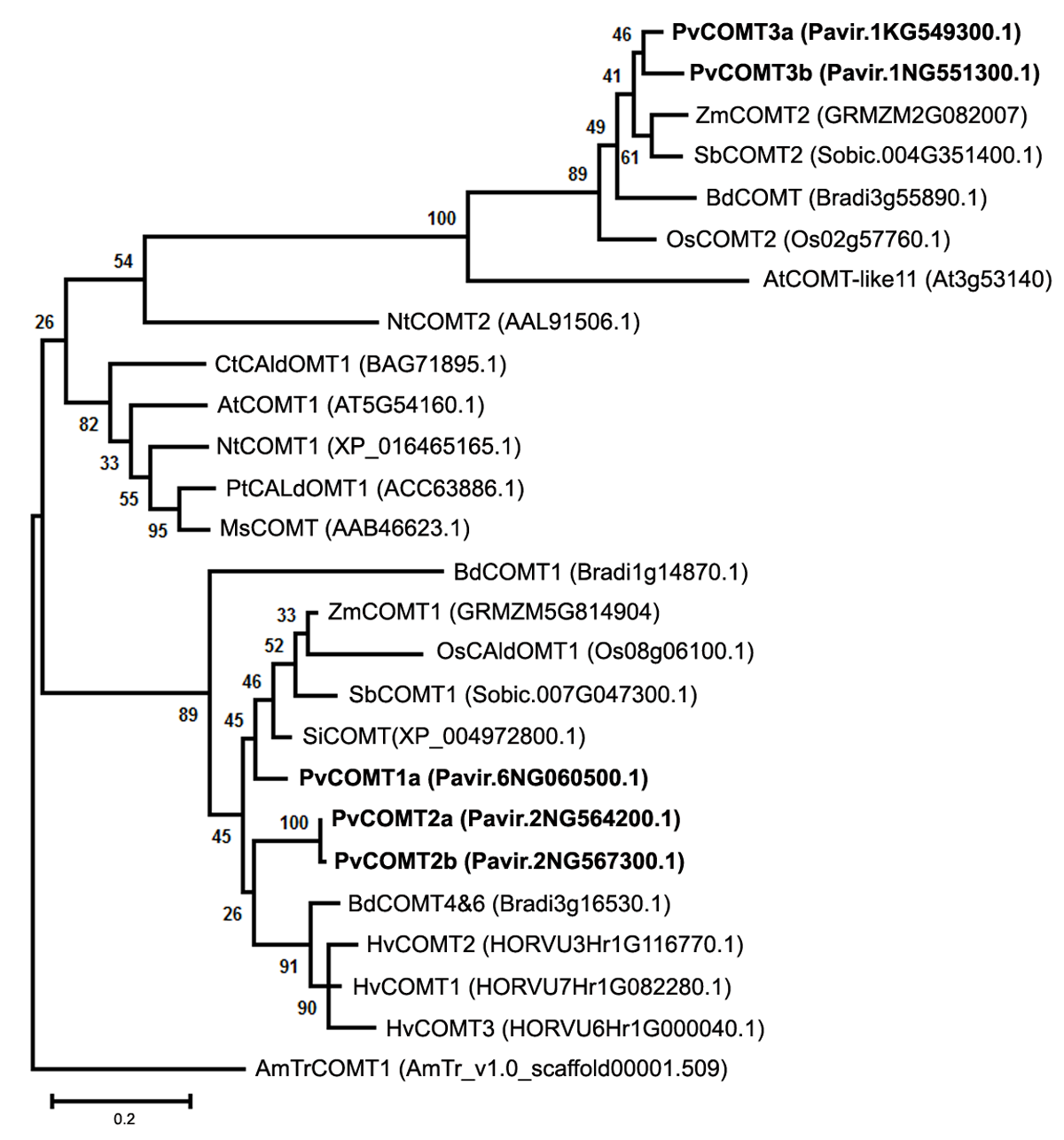


**Figure S2.** Phylogentic reconstruction of switchgrass caffeic acid 3-O-methyltransferase (COMT)amino acid domain sequences with other COMT proteins from the literature. The O-methyltransferase domain from each of the COMT sequences was identified using online MOTIF search tool (<https://www.genome.jp/tools/motif/>) and is given in Supplementary File 1. Phylogenetic analysis was conducted using the Maximum Likelihood method in MEGA X. The percentage of trees in which the associated sequences clustered together is shown next to the branches. Switchgrass COMTs are represented in bold font.


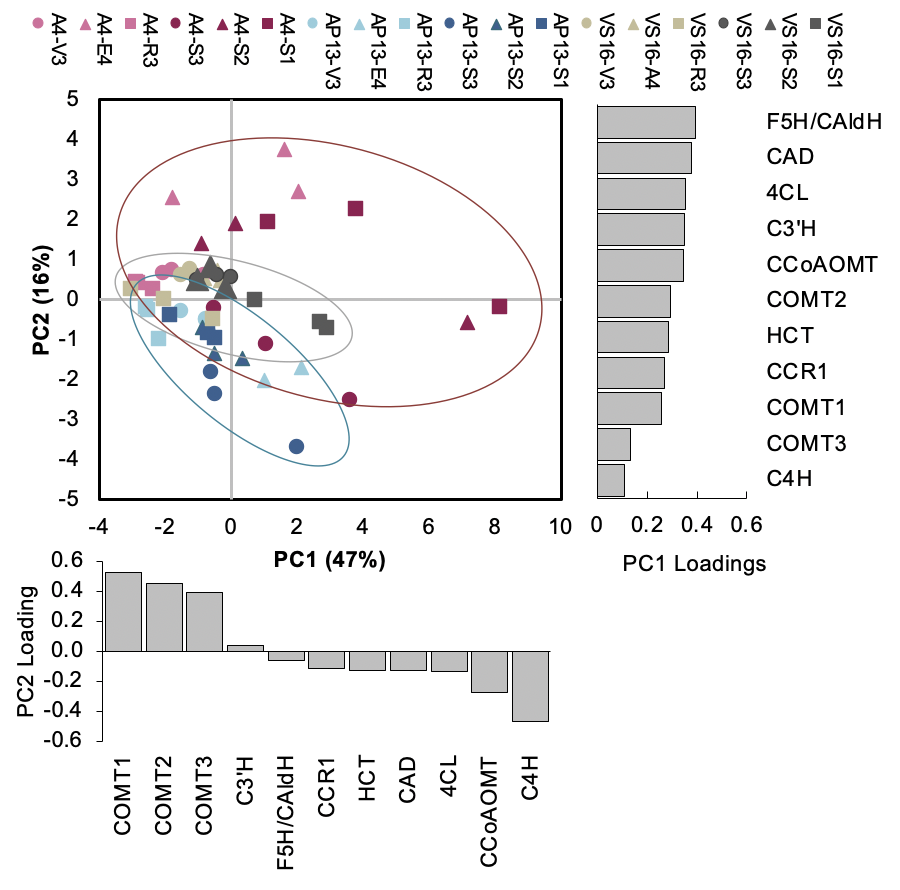


**Figure S3.** Principal component analysis of the expression of phenylpropanoid biosynthesis genes indicates large overlaps among samples types and genotypes. (main panel). The central graph indicates the distribution of samples in the first two principle components. Percentages indicate the variation represented by each component. Red indicates A4; blue AP13; grey VS16. Lighter shading indicates whole tillers and darker shading indicates segments of the elongation 4 (E4) tiller. Circles are younger, triangles intermediate, and squares older tillers/segments. Side panels indicate loadings of the two major principal components. *4CL*: *4-Coumarate:CoA Ligase*; *C3’H*: *para-Coumarate 3’-Hydroxylase*; *C4H*: *Cinnamate 4-Hydroxylase*; *CAD*: *Cinnamyl alcohol dehydrogenase*; *CCoAOMT*: *Caffeoyl CoA O-Methyltransferase*; *CCR*: *Cinnamyl CoA Reductase*; *COMT*: *Caffeic acid 3-O methyltransferase*; *F5H/Cald5H*: *Ferulate 5-hydroxylase/Coniferaldehyde 5-hydroxylase*; *HCT*: *para-Hydroxycinnamoyl-CoA:Quinate/Shikimate p-Hydroxycinnamoyltransferase*.

**
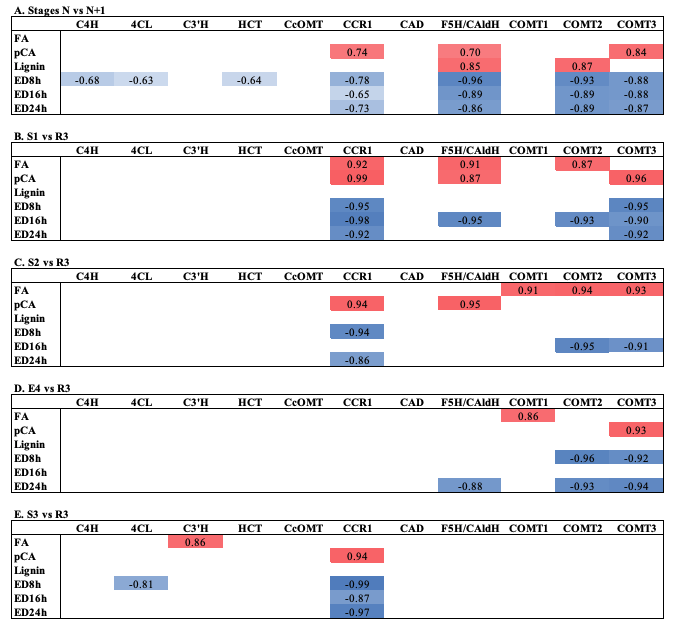
**

**Figure S4. C**orrelations between phenylpropanoid gene expression (GE) and absolute cell wall (CW) traitsfor highest precision models suggest that expression of *CCR1*, *F5H/CAldH* and *COMT*s in samples from mid-development are most indicative of phenylpropanoid accumulation and ED recalcitrance at maturity. (A) N_GE_ vs. N+1_CW_ model for the developmental stages (excluding segments). (B) Elongation4 (E4) S1_GE_ vs. Reproductive3_CW_(R3) model. (C) E4-S1_GE_ vs. R3_CW_ model. (D) E4_GE_ vs. R3_CW_model. (E)E4-S3_GE_ vs. R3_CW_ model. ED#h indicates the enzymatic digestibility yield after # hours of incubation. Color intensity represents magnitude of Gini Correlation Coefficient. Only correlations with*q*<0.01 are shown. Model statistics are provided in Table 3. Gene names are given in the legend of Figure S3. Correlation coefficients, *p-values*, and *q-values* are available in Table S4.

**Supplementary Data 1.** COMT domains

>PvCOMT1a

WYYLKDAVLEGGIPFNKAYGMTAFEYHGTDPRFNRVFNEGMKNHSVIITKKLLEFYAGFEGVGTLVDVGGGVGATLHAITSRYPGIRGVNFDLPHVISEAPPFPGVEHVGGDMFKAVPAGDAILMKWILHDWSDAHCAAILKNCYDALPAGGKVIAVECILPVNPEATPKAQGVFHVDMIMLAHNPGGKERYEREFEELAKGAG

>PvCOMT2a

WYYLKDAVLEGGIAFNKAYGMSAFEYHGTDPRFNRVFNEGMKNNSVIITKKLLESYTGFEGIGTLVDVGGGVGATIHAITSRYPHIKGINFDLPHVISDAPPFPGVEHVGGDMFKSVPSGDAIFMKWILHDWSDAHCATLLKNCYDALPAHGKVIVVECILPKNPEAIPKTQVMFHADMIMLAHNPGGKERYEREFEELVRGVGFK

>PvCOMT2b

WYYLKDAVLEGGIAFNKAYGMSAFEYHGTDPRFNRVFNEGMKNNSVIITKKLLESYTGFEGIGTLVDVGGGVGATIHAITSRYPHIKGINFDLPHVISDAPPPFPGVEHVGGDMFKSVPSGDAVFMKWILHDWSDAHCATLLKNCYDALPAHGKVIVVECILPKNPEAIPKTQVMFHADMIMLAHNPGGKERYEREFEELVRGVGFK

>PvCOMT3a

WPRLHEAVLDPAGPEPFARANAGVPAYAYYGKDREANQVMLRAMTGVSEPFMDALLDGYAQGFHGVATLVDVGGSSGACLQMIMRRVATITEGINFDLPDVVAAAPPIAGVRHVGGDMFKSIPSGDAIFMKWVLTTWTNDECTAILRNCHSALPAGGKVIACEPVVPEETDTSTRTRALLENDIFVMTTYRTQGRERSEDEFRRLGLAAGF

>PvCOMT3b

RAMTGVSEPFMEALLDGYAGGFDGVATLVDVGGSSGACLEMIMGRVGTITEGINFDLPNVVAAAPPIAGVRHVGGDMFKSIPSGGAIFMKWVLTTWTNDECTAILRNCHSALPAGGKLIACEPVVPEETDTSTRTRALLENDIFVMTTDRTLGRERSEDEFRSLGLATG

>AtCOMTlike

TAVVEPETEPYVKANGEAAYAQYGKSEEMNGLMQKAMSGVSVPFMKAILDGYDGFKSVDILVDVGGSAGDCLRMILQQFPNVREGINFDLPEVVAKAPNIPGVTHVGGDMFQSVPSADAIFMKWVLTTWTDEECKQIMKNCYNALPVGGKLIACEPVLPKETDESHRTRALLEGDIFVMTIYRTKGKHRTEEEFIELGLSAGF

>SeitaCOMT

WYYLKDAVLEGGIPFNKAYGMTAFEYHGTDPRFNRVFNEGMKNHSVIITKKLLEFYTGFEGVGTLVDVGGGIGATLHAITSCHPQIKGVNFDLPHVISEAPPFPGVQHVGGDMFKSVPAGDAILMKWILHDWSDAHCATILKNCYDALPANGKVIIVECVLPVNPEATPKAQGVFHVDMIMLAHNPGGKERYEREFRELAKGAGF

>SbCOMT1

WYYLKDAVLDGGIPFNKAYGMTAFEYHGTDPRFNRVFNEGMKNHSVIITKKLLEFYTGFDESVSTLVDVGGGIGATLHAITSHHSHIRGVNFDLPHVISEAPPFPGVQHVGGDMFKSVPAGDAILMKWILHDWSDAHCATLLKNCYDALPEKGGKVIVVECVLPVTTDAVPKAQGVFHVDMIMLAHNPGGRERYEREFRDLAKAAGFS

>SbCOMT2

WPRLHEAVLDPRGPEPFARANAGVPAYAYYGKDREANQVMLRAMTGVSEPFMHALLDGYGFDGGFHGVATLVDVGGSSGACLEMIMRRVPTIKEGINFDLPDVVADAPAIAGVRHVGGDMFKSIPSGDAIFMKWVLTTWTNDECTAILRNCHAALPDGGKLVACEPVVPEETDSSTRTRALLENDIFVMTTYRTQGRERSEEEFHHLGIAAG

>ZmCOMT1

WYYLKDAVLDGGIPFNKAYGMTAFEYHGTDSRFNRVFNEGMKNHSVIITKKLLDFYTGFEGVSTLVDVGGGVGATLHAITSRHPHISGVNFDLPHVISEAPPFPGVRHVGGDMFASVPAGDAILMKWILHDWSDAHCATLLKNCYDALPENGKVIVVECVLPVNTEATPKAQGVFHVDMIMLAHNPGGKERYEREFRELAKGAGFS

>ZmCOMT2

WPRLHEAVLDPAGPEPFARANAGVTAYAYYGKDQDANRVMLRAMAGVSEPFMGALLDGYGAAGGFRGVATLVDVGGSSGACLEMIMRRVPTITEGINFDLPDVVAAAPPIAGVRHVGGDMFKSIPSGDAIFMKWVLTTWTDDECTAILRNCHAALPDGGKLVACEPVVPEETDSSTRTRALLENDIFVMTTYRTQGRERSEEEFRHLGVDAAGF

>OsCAldOMT1

WYYLKDAVLDGGIPFNKAYGMTAFEYHGTDARFNRVFNEGMKNHSVIITKKLLDLYTGFDAASTVVDVGGGVGATVAAVVSRHPHIRGINYDLPHVISEAPPFPGVEHVGGDMFASVPRGGDAILMKWILHDWSDEHCARLLKNCYDALPEHGKVVVVECVLPESSDATAREQGVFHVDMIMLAHNPGGKERYEREFRELARAAGF

>OsCOMT2

WPLLHEAVLDPSGPEPFARANAGVPAYAYYGKDREANEVMLRAMTGVSEPFMEALLEGYGDGGFEGVSTLVDVGGSSGACLEMIMRRVRTIRDGVNFDLPDVVAAAPPIPGVRHVGGDMFKSIPSGDAIFMKWVLTTWTNEECTAILSNCHKALPGGGKVIACEPVVPDTTDGSTRTRALLENDIFVMATYRTQGRERSEEEFRHLGLAAGF

>BdCOMT46

WYYLKDAVLDGGIPFNKAYGMSAFEYHGTDPRFNRVFNEGMKNHSIIITKKLLDLYPGFEGLGTLVDVGGGVGATVGAIVARHPAIKGINFDLPHVISEGIPFPGVTHVGGDMFQKVPSGDAILMKWILHDWSDAHCATLLKNCYDALPAHGKVVIVECILPVNPEATPKAQGVFHVDMIMLAHNPGGKERYEREFEELARGAGF

>BdCOMT1

WYHLKDVVLDGGLPFNKAHGIIAFEYHGKDARFDRVFNEAMKNHSTILTKKFLEFYTGFDDVKTLVDVGGGVGATIRAIISKYPHISGVNFDLPHVISSAPTCPGVQHIGGDMFKKVPSGDAILMKWILHDWTDDHCMMLLRNCYDALPVGGKLIIIESILPVNPEATPRARMAFEDDMIMLTYTPGGKERYKREFEVLAKGARF

>BradiCOMT

WPRLHEALLDPAGPEPFARAHRGLPAYAFYAQDKEANEVMLRGMTGVSEPFMEALLDGYAGGFEDVRTLVDVGGSSGACLDMIMRRVGTIAQGINFDLPDVVAAAPPIAGVRHVGGDMFKSIPSGDAIFMKWVLTTWTNDECTAILKNCYGALPEGGKLIACEPVVPETTDTSTRTRALLENDIFVMTTYRTQGRERSEEEFRQLGLAAGF

>AtCOMT1

WYHLKDAILDGGIPFNKAYGMSAFEYHGTDPRFNKVFNNGMSNHSTITMKKILETYKGFEGLTSLVDVGGGIGATLKMIVSKYPNLKGINFDLPHVIEDAPSHPGIEHVGGDMFVSVPKGDAIFMKWICHDWSDEHCVKFLKNCYESLPEDGKVILAECILPETPDSSLSTKQVVHVDCIMLAHNPGGKERTEKEFEALAKASGFK

>PtCALdOMT1

WYYLKDAILDGGIPFNKAYGMTAFEYHGTDPRFNKVFNKGMSDHSTITMKKLLETYKGFEGLTSLVDVGGGTGAVVNTIVSKYPSIKGINFDLPHVIEDAPSYPGVEHVGGDMFVSVPKADAVFMKWICHDWSDAHCLKFLKNCYDALPENGKVILVECILPVAPDTSLATKGVVHIDVIMLAHNPGGKERTEKEFEGLAKGAGFQ

>HvCOMT1

WYYLKDAVLDGGIPFNKAYGMSAFEYHGTDPRFNRVFNEGMKNHSIIITKKLLEVYKGFEGLGTIVDVGGGVGATVGAITAAYPAIKGINFDLPHVISEAPPFPGVTHVGGDMFQKVPSGDAILMKWILHDWSDEHCATLLKNCYDALPAHGKVVLVECILPVNPEATPKAQGVFHVDMIMLAHNPGGRERYEREFEALAKGAGF

>HvCOMT2

WYYLKDAVLDGGIPFNKAYGMSAFEYHGTDPRFNRVFNEGMKNHSIIITKKLLESYKGFEGLGTLVDVGGGVGATVGAIIARYPAVKGINFDLPHVISEAPAFPGVTHIGGDMFQKVPSGDAILMKWILHDWSDEHCATLLKNCYDALPAHGKVVLVECILPVNPEATPEVQGVFHVDMIMLAHNPGGRERYEREFEALAKGAGF

>HvCOMT3

WYYLKDAVLDGGIPFNKAYGMSAFEYHGTDPRFNRVFNEGMKNHSIIITKKLLELYKGFNGLSTLVDVGGGIGATVGAITAYYHTIKGINFDLPHVISEAPLFPGVTHVGGDMFQKIPSGDAILMKWILHDWSDEHCATLLKNCYDALPAHGKVVLVECILPVNPEATPKAQGVFNLDMIMLAHNPGGRERYEREFEALAKGGGF

>NtCOMT1

WYHLKDAVLDGGIPFNKAYGMTAFEYHGTDPRFNKVFNRGMSDHSTMSMKKILEDYKGFEGLNSIVDVGGGTGATVNMIVSKYPSIKGINFDLPHVIGDAPTYPGVEHVGGDMFASVPKADAIFMKWICHDWSDEHCLKFLKNCYEALPANGKVIIAECILPEAPDTSLATKNTVHVDIVMLAHNPGGKERTEKEFEALAKGAGF

>NtCOMT2

WFELKDAVLEGGVPFDRVHGVHAFEYPKSDPKFNDVFNKAMINHTTVVMKKILENYKGFENLKTLVDVGGGLGVNLKMITSKYPTIKGTNFDLPHVVQHAPSYPGVEHVGGDMFESVPEGDAIFMKWILHDWSDSHNLKLLKNCYKALPDNGKVIVVEAILPVKPDIDTAVVGVSQCDLIMMAQNPGGKERSEEEFRALATEAGFK

>CtCAldOMT1

WYHLKDAVLDGGIPFNKAYGMSAFEYHGKDERFNKVFNSGMFNHSTMTMKKILDVYPGFNGVKTLVDVGGGTGASLSMILSKHPSIKGINFDLPHVIQDATNYPGIEHVGGDMFESVPKGDAIFMKWICHDWSDAHCLKFLKNCYKALPENGKVIVAECILPETPDSSLATKNVVHIDVIMLAHNPGGKERTEKEFQALAKAAGFK

>MsCOMT

WYHLKDAVLDGGIPFNKAYGMTAFEYHGTDPRFNKVFNKGMSDHSTITMKKILETYTGFEGLKSLVDVGGGTGAVINTIVSKYPTIKGINFDLPHVIEDAPSYPGVEHVGGDMFVSIPKADAVFMKWICHDWSDEHCLKFLKNCYEALPDNGKVIVAECILPVAPDSSLATKGVVHIDVIMLAHNPGGKERTQKEFEDLAKGAGFQ

>AmTrCOMT1

WYHLKDAVLEGGVPFNRAYGMTAFEYHGKDPRFNKVFNRGMSDHSTITMKKILDDYPGFEGLNCIVDVGGGIGATLNMIVGKYGHIKGINFDLPHVIADAPEFPGVTHVGGDMFESVPTGDAIFMKWILHDWGDEYCHKLLKNCYKALPDSGKVIIAESILPIAAENSLAAHGVFHVDCIMLAHNPGGKERTEKEFEALAKGAGF
